# The diagnostic power of cervico-vaginal fluid prolactin in the diagnosis of premature rupture of membranes

**Published:** 2012-09-30

**Authors:** N Kariman, M Hedayati, Sh Alavi Majd

**Affiliations:** 1 Nursing Midwifery Faculty, Shahid Beheshti Medical University, Tehran, Iran; 2 Obesity Research Center, Research Institute for Endocrine Sciences, Shahid Beheshti Medical University, Tehran, Iran; 3 Paramedicine Faculty, Shahid Beheshti University of Medical Sciences, Tehran, Iran

**Keywords:** Premature rupture of membranes, Prolactin, Vaginal washing fluid

## Abstract

**Background:**

Premature rupture of membranes (PROM) is one of the most common complications of pregnancy that has a major impact on pregnancy outcomes. A diagnostic tool that is non-invasive, specific and quick is needed to predict PROM. The purpose of this study was to evaluate the diagnostic power of the vaginal washing fluid prolactin for the diagnosis of premature rupture of membranes and to determine cut-off values.

**Methods:**

A total of 114 pregnant women were recruited in this diagnostic trial. The PROM group consisted of 54 pregnant women between 20 and 41 weeks of gestation with diagnosis of confirmed PROM [amniotic fluid pooling (+) and Nitrazine paper test (+) and fernt test (+)]. The control group consisted of 60 pregnant women between 20 and 41 weeks of gestation without any complaint or complication. All patients underwent speculum examination for amniotic fluid pooling, nitrazine paper test, fern test, vaginal washing fluid prolactin sampling.

**Results:**

Vaginal fluid concentrations of prolactin was significantly different between the two groups (P < 0.001). The sensitivity, specificity, positive and negative predictive values and accuracy were 87.03%, 75.0%, 75.80%, 86.53% and 83.33% in detecting PROM by evaluation of vaginal fluid prolactin concentration with a cut-off value of 9.50 µIU/ml, respectively.

**Conclusions:**

The prolactin levels in the washing fluid of the posterior vaginal fornix in our experience is reliable and non-invasive diagnostic tests of PROM.

## Introduction

Premature rupture of membranes (PROM) refers to rupture of the fetal membranes prior to the onset of labor (1) and can occur at any gestational age even at 42^nd^ week. (2,3) PROM has previously been reported to occur in 8-19.53% of term pregnancies (2,4,5) and 2-25% of all pregnancies. (6) Besides, Nili and Shams Ansari (7) reported a prevalence of 7% in Vali-e-Asr hospital of Tehran. 

PROM has been shown to be the cause of 18 to 20 % of prenatal mortalities (4) and 21.4% of prenatal morbidity. (8-10) Compared with normal group, the average hospitalization period of term and preterm newborns with PROM were prolonged 20% and 25.1% respectively. Consequently, the average costs of hospitalization were increased 30.5% and 60% respectively. (10) Maternal complications include clinically evident intra-amniotic infection which occurs in 13% to 60% of women with PROM in comparison with 1% prevalence of term and postpartum endometritis. (5,11) PROM is a clinical diagnosis actually. It is typically suggested by a history of watery vaginal discharge and is confirmed on sterile speculum examination.

The traditional minimally invasive gold standard for diagnosis of PROM relies on clinician’s ability to document three clinical signs on sterile speculum examination: [1] visual pooling of clear fluid in the posterior fornix of the vagina or leakage of the fluid from the cervical os; (12) an alkaline pH of the cervico-vaginal discharge, which is typically demonstrated by nitrazine paper; and/or (2) microscopic ferning of the cervico-vaginal discharge. (2,3,13) Diagnosis of PROM is easy in the presence of obvious rupture of membranes while several numbers of false positive and negative results obtained through applying conventional diagnostic methods in the suspected cases of PROM may result in inappropriate interventions such as hospitalization and induction of labor. On the other hand, misdiagnosis of PROM may divert the patient from appropriate treatments. (14-17) Although inspection of fluid leakage from cervix has been traditionally the only method for definite diagnosis of PROM, it is associated with 12 to 30% false negative results. Intermittent or low volume vaginal discharge or presence of urine or semen may interfere with diagnosis of PROM. Nitrazine and fern tests may also lead to false positive or negative results. (4,18)

Several studies have been conducted to find a definite, easy, noninvasive and reliable diagnostic test for PROM in recent years. These studies have mainly focused on biochemical agents with high concentration in amniotic fluid. Prolactin, (19-21) alpha-fetoprotein (AFP), (19-22) insulin like growth factor binding protein (IGFBP-1), (23-26) fetal fibronectin (fFN), (27) Lactat, (7,28) beta-subunit of human gonadotropin (B-HCG) (14,15,29,30) and urea-creatininehave been mentioned as some of these factors. However, results of using aforementioned tests have been variable. (31,32)

Prolactin (PRL) is a 199-aminoacid single polypeptide chain and known as a lactogenic hormone. PRL is encoded by a single gene located on the short arm of chromosome 6 (34) During pregnancy PRL is produced by the maternal and fetal hypophyses and the decidua. (35) PRL concentrations rise steadily in maternal blood throughout pregnancy to about 10 times the non-pregnant value. PRL of amniotic fluid is five to 10 times higher than that of either maternal blood. (36) Thus we hypothesized that vaginal fluid PRL may be helpful in diagnosis of PROM. Indeed, the aim of this study was to evaluate the power diagnostic of vaginal washing fluid PRL for diagnosis of PROM and to determine cut-off values.

## Materials and Methods

This diagnostic trial study has been performed to evaluate a diagnostic test for PROM between November 2010 and July 2011 in Taleghani Hospital, prenatal clinic and delivery ward. Among 118 pregnant women who were admitted with the complaint of vaginal fluid leakage between 20 and 41 weeks of gestation, 54 cases with confirm PROM were included in the present study. The remaining pregnant women were excluded due to the visible blood in vaginal secretion, use of vaginal drugs or intercourse in the prior night, meconium in amniotic fluid, presence of fetal anomalies, intrauterine fetal death, known disease, prenatal complication, multiple pregnancies, suspicious PROM and regular uterine contractions. Demographic and obstetric characteristics, results of speculum examination, fern test, nitrazine test (MACHEREY-NAGEL GmbH & Co., Germany) and prolactin (Direct ELISA kit-Prolactin, Diagnostic Blochem Co., Canada) were documented according to a data form, validity of which was confirmed by content validity method. Prolactin concentration was measured by ELISA method. Control solutions were used to confirm validity of ELISA method. The reliabilities of data form and speculum physical exam were confirmed by test-retest and reliability of ELISA, fern and nitrazine tests were established by inter-rater consistency.

This study was approved by ethics committee of Shahid Beheshti Medical University and written informed consent was obtained from all participants. Gestational age was determined based on the first day of last menstruation period in reliable cases, or one ultrasound in less than 14 weeks or two ultrasound documents between 14 and 24 weeks of pregnancy. Pregnant women were examined in lithotomy position, leakage of fluid was inspected by sterile speculum and results were registered as positive, negative or suspicious. A cotton tip applicator was inserted in deep vagina and was immediately transferred on nitrazine paper. PH above 6.5 was considered positive. A sample of cervicovaginal secretion was taken by a similar method and was expanded on slides. The slides were examined after drying by microscope (10× magnification) for diagnosis of ferning pattern. Patients who had positive pooling, nitrazine paper test and fern test were considered as confirmed PROM group. Furthermore, diagnosis of PROM was confirmed by AFI (Amniotic Fluid Index) through ultrasound examination. Meanwhile, among pregnant women admitted to prenatal clinic for their regular prenatal control visit, 60 pregnant women with 20 to 41 weeks of gestational age without any complaint or complication and with pooling (-), nitrazine paper test (-) and fern test (-) were taken as control group. Procedures described before were applied to patients of control group as well. Thereafter, vaginal washing fluid prolactin sampling was performed as follows: Three ml of sterile normal saline was injected into the posterior fornix of vagina and then was aspirated by the same syringe and was sent immediately to the laboratory. All speculum examinations were performed by the same obstetrician and all samples were studied in Research Institute for Endocrine Sciences Laboratory (which is located in Taleghani Hospital) and by the same technique in order to eliminate inter-observer sampling difference. Cut-off value was determined by receiver operating characteristic (ROC) curve. Statistical analysis was performed by SPSS (v.18) software. Results have been expressed as frequency, mean and standard deviation. We conducted Chi (2) test on education, job and Mann Withney test on gravida and parity. The parametes of age and gestational age were compared with T-test. P value less than 0.05 was considered statistically significant.

## Results

Demographic data for each group are presented in Table 1... The proportion of high school education was highest among educational degrees in the groups (46.3%=PROM and 41.70%=control group, P=0.41). Most of the patients were housewives (PROM=90.70% and control=93.3%, P=0.31). No statistically significant difference has been observed between these groups with respect to these factors.

**Table 1:** The demographic characteristics of groups^a^

****** ******	**(PROM)** **(n=54)** **X±SD**	** (Intact membranes)** **(n=60)X±SD**	**P **
Age(year)	25.40±5.54	26.05±5.20	0.52
Gestational age (week)	38.15±0.34	38.07±0.44	0.84
Gravida	1.74±1.40	1.83±1.16	0.65
Parity	0.56±0.13	0.60±0.11	0.88

^a^Difference between groups tested with T- test (age and gestational age) and Mann-Whitney U test (gravida and parity).

Table 2 shows the vaginal fluid prolactin levels (µIU/ml) among groups. The mean concentration vaginal fluid prolactin levels in the PROM group was 851.22±425.74 _µIU/ml_ (range 5.00-5551). This is significantly (p<0.001) higher than the value obtained for control group (i.e., 8.20±0.67_µIU/ml_, range 4.00-24.00).

**Table 2:** Vaginal fluid prolactin levels (µIU/ml) among groups

**	** (PROM)** **(n=54)** **X±SD**	** ( Intact membranes)** **(n=60)X±SD**	**P **
Prolactin ( µIU/ml )^a^	851.22±425.74	8.20±0.67	<0.001

^a^Difference between PROM and intact membranes tested with T- test.

**Figure 1:**
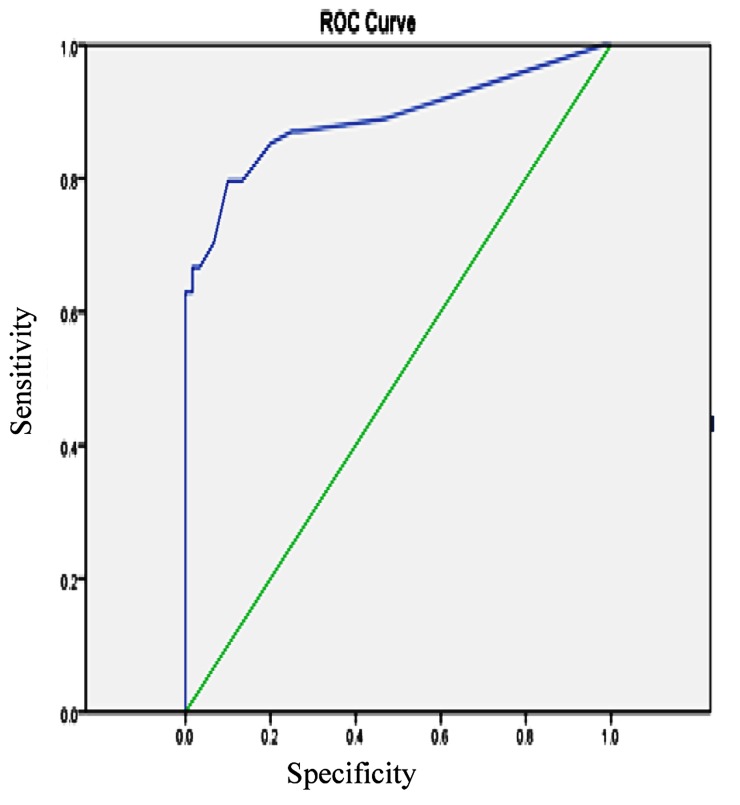
Receiver operating characteristic curve for vaginal prolactin Levels

ROC curve analysis was used to establish the optimal cut-off concentrations for vaginal washing fluid prolactin. From the ROC curves, 9.50_µIU/ml_ was set as a cut-off value for prolactin (Fig 1). The area under the curve is 89.90% for prolactin. According to the prolactin cut-off point sensitivity of 87.30%, specificity of 75.0%, positive predictive value of 75.80%, negative predictive value of 86.53% and accuracy of 83.33% were found respectively.

## Discussion

As mentioned before, a timely and accurate diagnosis of PROM is therefore critical to optimize perinatal outcome and minimize serious complications such as cord prolapse and infections including chorioamnionitis and neonatal sepsis. (19,29,30,37) In most cases diagnosis is made according to the clinical complaints and traditional methods. (14) However, clinical complaint of patient is not reliable. (15)

With the possible exception of direct visualization of amniotic fluid spurting from the cervical os, all clinical signs have limitations in terms of diagnostic accuracy, cost and technical case. Moreover, reliance on clinical assessment alone leads to both false-positive and false–negative results. (2) Thus, we need simple, reliable and rapid tests for diagnosis of PROM. Since there is no unique and noninvasive gold standard test applicable to all patients with 100% accuracy several biochemical markers have been studied previously. (15) Despite the improved diagnostic value of these markers, they have not become popular because of their complexity and cost. (14)

This study showed that diagnostic power of PRL for PROM was in acceptable range. As far as we know, limited studies related to PROM and vaginal washing fluid PRL have been published so far. One of these studies was conducted by Shahin and Raslan (2007). The purpose of this study was to determine the effectiveness of vaginal fluid BhCG, AFP and PRL measurements in detection of PROM. The results showed that vaginal fluid concentrations of three markers were significantly higher in the PROM group than in the control group. A cut-off value of 20.2 _µIU/ml_ was proposed for PRL and its sensitivity, specificity, positive predictive value, negative predictive value and efficacy of PRL were 70%, 76%, 71.7%, 74.5%, and 73%, respectively. (38)

The second study was carried out by Buyukbayrak etal. (2004). In that study 38 patients with confirmed PROM, 32 patients with suspected but unconfirmed PROM and 70 pregnant women without any complaint or complication were included. The sensitivity, specificity, positive predictivity and negative predictivity were 95%, 78%, 93%, 84%, and 87%, respectively in detecting PROM by evaluation of vaginal PRL concentration with cut-off values of 30_µIU/ml_. (18) 

In Phocas etal. research (1989), maternal serum and vaginal fluid values of AFP, PRL and hPL (human placental lactogen) were measured. The study group consisted of 21 women with the diagnosis of PROM while the control group consisted of 12 pregnant women with intact membranes. They concluded that in PROM, vaginal fluid PRL levels were significantly higher (2-10 fold) than the paired maternal serum PRL and ranged from 130-2315_ng/ml_. In contrast, vaginal PRL and urine PRL concentrations in pregnancies without PROM were very low or undetectable (range: 0-5_ng/ml_ and 0.15-1_ng/ml_, respectively. (39) 

Our results are in good agreement with these three studies. Shahin etal. and buyukbarak etal. utilized the ECLICA method (Electrochemoluminescence assay) to measure PRL that is more sensitive than ELISA. However, it is an expensive and complex test. Furthermore, it is not available in most laboratories. An ideal laboratory diagnostic technique should be affordable and available. 

In contrast, Huber etal. assayed the amount of PRL, AFP and hPL in vaginal washing fluid. Despite the higher concentration levels of the three markers in PROM group, they speculated that, measurement of these proteins in vaginal fluid could not be a suitable clinical test for the diagnosis of PROM. The reason was the presence of considerable overlap between groups and a high rate of false-positives. 

In the present study using ELISA method, we determined a cut-off value of 9.50_µIU/ml_ for PRL. We have found that power diagnostic including sensitivity, specificity, positive predictive value, negative predictive value and accuracy of vaginal fluid PRL were 87.30%, 75.0%, 75.80%, 86.53% and 83.33%, respectively. Our study reported low vaginal PRL in pregnant women with intact amniotic membranes (control group). After rupture of fetal membranes a high level of PRL can be detected in vaginal fluid discharge. Meanwhile, since the concentration of PRL is always higher in amniotic fluid than in maternal serum and urine, the test for PRL can be reliable and helpful even in the presence of vaginal bleeding or urine. 

In current study, three tests including direct speculum examination, fern test and nitrazine test were applied for diagnosis of PROM. Furthermore, inclusion criteria were so that interfering factors of these tests could be controlled. 

In conclusion, our study demonstrated that the measurement of vaginal fluid PRL with ELISA method is a reliable test for diagnosis of PROM.
